# Epidemiology and treatment patterns of essential tremor: a retrospective cohort analysis in Germany

**DOI:** 10.3389/fneur.2025.1580919

**Published:** 2025-07-02

**Authors:** Jos S. Becktepe, Keltie McDonald, Sabrina Müller, Thomas Wilke, Evi Zhuleku, Karen Appiah, Natasha Dzimitrowicz, Jade Marshall, Javier Sabater, Luigi M. Barbato, Tabish A. Saifee

**Affiliations:** ^1^Department of Neurology, Christian-Albrechts-University, Kiel, Germany; ^2^Cytel, London, United Kingdom; ^3^IPAM e.V. (Institute for Pharmacoeconomics and Drug Logistic), Wismar, Germany; ^4^Cytel, Berlin, Germany; ^5^Jazz Pharmaceuticals, London, United Kingdom; ^6^Jazz Pharmaceuticals, Palo Alto, CA, United States; ^7^National Hospital for Neurology and Neurosurgery, London, United Kingdom

**Keywords:** essential tremor, epidemiology, real-world evidence, real-world treatment, administrative claims data

## Abstract

**Background:**

Real-world evidence on the epidemiology, clinical characteristics, and treatment patterns of patients with essential tremor (ET) is limited. We used data from two large representative German claims databases to address this evidence gap.

**Methods:**

Data were obtained from two German statutory health insurance databases, AOK PLUS and GWQ ServicePlus, from January 1, 2010 to March 31, 2022. Point prevalence and cumulative incidence of ET, standardized by age and sex to the German national population, were assessed. Baseline demographics, clinical characteristics, and treatment patterns were described within a cohort of patients newly diagnosed with ET in each database. Time to treatment initiation from diagnosis, and discontinuation and switch from the first-line therapeutic approach were evaluated using Kaplan Meier methods.

**Results:**

Age and sex-standardized prevalence of ET increased between 2010 and 2021, reaching 196 (AOK) and 250 (GWQ) per 100,000 persons in 2021. Among patients newly diagnosed with ET, the most frequent comorbidities at baseline were pain disorders (65–70%), hypertension (44–65%), and hyperlipidaemia (30–35%). Approximately 60% of patients received pharmacological therapy during follow-up (mean 56–62 months), particularly propranolol (44–50%), bisoprolol (24–27%) and metoprolol (23–27%). The median time from diagnosis to treatment initiation was 2.1–6.3 months. Most patients discontinued (72–75%) their first therapy within 12 months, with 41–46% switching to another therapy.

**Conclusion:**

ET is a common movement disorder, the management of which is multi-faceted. Further evidence is needed to better understand and prioritize unmet needs and improve outcomes for patients with ET.

## 1 Introduction

Essential tremor (ET) is a neurologic condition characterized by a bilateral action tremor of the upper limbs. The condition is often asymmetric and may present with or without tremor in other locations like the head, voice, and lower limbs (1). ET shows variations in age of onset, comorbid conditions, and response to treatment (2). Symptoms of ET tend to be chronic and progressive and can significantly impact quality of life and ability to perform daily activities (3).

While few studies have evaluated the incidence of ET, existing epidemiological evidence suggests that it is one of the most common movement disorders. A recent meta-analysis estimated that ET affects approximately 1.3% of the population worldwide (4). Based on primary care data from France and the United Kingdom (UK), the annual incidence rate of ET is approximately 18–21 per 100,000 person-years, and incidence tends to increase with age, from approximately 4 per 100,000 person-years in those under 20 years to over 51 per 100,000 person-years in those aged 80 years and older (5). Importantly, evidence suggests that the incidence of ET may be underreported due to undertreatment (6) and misdiagnosis (7). Furthermore, while reported epidemiological estimates are heterogeneous, to our knowledge, no studies of the epidemiology of ET have been conducted in Germany to date.

Treatment for ET aims to minimize the impact of the tremors on an individual’s daily life, with pharmacotherapy as the most common approach. In particular, propranolol, primidone, and topiramate are considered first-line options with the highest level of evidence (8). Moreover, botulinum toxin (Botox) has also been shown to reduce tremors in selective muscles (9). In addition to pharmacological therapies, other medical interventions including deep brain stimulation and focused ultrasound have also shown promise in providing symptomatic relief to patients with ET (10–13). Despite their availability, existing pharmacological treatment is effective in only approximately half of patients with ET, reflecting a large unmet need (14). Furthermore, many treatments have adverse effects that need to be weighed against their benefits (14, 15).

Given the burden of ET and considerable unmet need, further evidence is needed to better understand its epidemiology and the characteristics of patients diagnosed and treated in the real world. This study used secondary data from two large, representative administrative claims databases to investigate the epidemiology, clinical characteristics, and treatment of patients with ET in Germany.

## 2 Methods

### 2.1 Data source

This retrospective study was conducted using anonymized German statutory health insurance (SHI) data provided by AOK PLUS (AOK) and GWQ ServicePlus (GWQ), covering the period from January 1, 2010 to March 31, 2022. AOK insures approximately 3.4 million persons in the regions of Saxony and Thuringia (central-eastern Germany), corresponding to 50% of the local population. The GWQ research database includes records from approximately 5 million insured persons from smaller sickness funds across Germany. AOK and GWQ are mutually exclusive databases, together covering approximately 11–12% of the SHI population in Germany. Due to the regulations for data use, data from the two sickness funds cannot be combined at patient-level and results in this study were investigated and presented separately.

### 2.2 Study population

Epidemiology of ET including point prevalence (2010–2021) and cumulative incidence (2011–2021) was evaluated across the study period in each calendar year. To assess patient characteristics and treatment utilization, a cohort of newly diagnosed patients was identified. Specifically, the study population consisted of patients with newly diagnosed ET based on the presence of one inpatient and/or two outpatient confirmed ET diagnoses [International Statistical Classification of Diseases and Related Health Problems, 10th revision, German Modification (ICD-10-GM) code: G25.0] made by any specialty in two different quarters within a period of 12 months in the inclusion period between January 1, 2011 to March 31, 2021. The inclusion period allowed for a 12-month pre-index period, ensuring the identification of incident ET diagnoses and the description of baseline characteristics, and a 12-month minimum follow-up period. Patients were followed from the index date to the earliest of end of insurance, death, or end of the study period (March 31, 2022).

**Figure 1:**
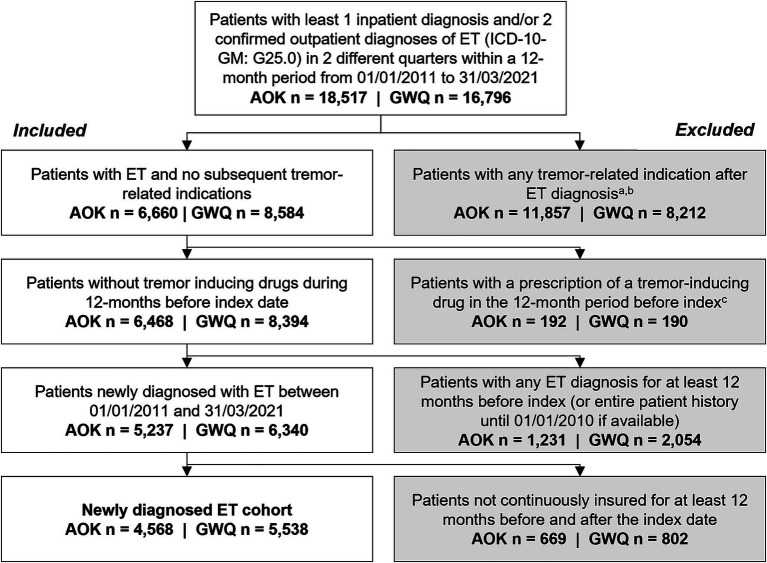
Attrition chart for selection of newly diagnosed patients with ET in AOK and GWQ.a. Including abnormalities of gait and mobility (ICD-10-GM: R26), Parkinson’s disease (ICD-10-GM: G20), drug-induced tremor (ICD-10-GM: G25.1), dystonia (ICD-10-GM: G24); multiple sclerosis (ICD-10-GM: G35), Huntington’s disease (ICD-10-GM: G10), other specified forms of tremor (ICD-10-GM: G25.2), myoclonus (ICD-10-GM: G25.3), inflammatory neuropathy (ICD-10-GM: G61), hyperthyroidism (ICD-10-GM: E05), hyperparathyroidism (ICD-10-GM: E21.0-E21.3), Wilson disease (ICD-10-GM: E83.0), Parkinsonism/atypical Parkinsonism (ICD-10-GM: G21, G22, G23.1-G23.3). b. Three patients were excluded from the AOK cohort because diagnosis occurred during a non-insured period. c. Including valproate (ATC: N03AG01), lithium (ATC: N05AN01), cyclosporin (ATC: L04AD01), or tacrolimus (ATC: L04AD02). ATC, anatomical therapeutic chemical; ICD-10-GM, International Statistical Classification of Diseases and Related Health Problems 10th Revision-German modification; ET, essential tremor.

The index date was the date of the first ET diagnosis. To increase specificity of an ET diagnosis and avoid misclassification, patients were excluded if they had other tremor-related neurologic disorders or diseases with common symptomatic tremor (including abnormalities of gait and mobility, dystonia, multiple sclerosis, Huntington’s Disease, other specified forms of tremor, myoclonus, inflammatory neuropathy, hyperthyroidism, hyperparathyroidism, Wilson disease, and Parkinsonism/atypical Parkinsonism) after the first observed ET diagnosis (index) until the end of the study period. Furthermore, patients with any prescriptions of common tremor-inducing agents (valproate, lithium, cyclosporin, or tacrolimus) in the 12-month pre-index period were excluded (Figure 1).

### 2.3 Measures

#### 2.3.1 Baseline characteristics

Baseline characteristics including age, gender, year of initial ET diagnosis (index year), time from index date to first pharmacological ET prescription, Charlson Comorbidity Index score (CCI, Supplementary Table 1) (16), Elixhauser comorbidity index score (17), CHAS_2_DS_2_-VASc score (Supplementary Table 2) (18–20), and pre-specified comorbidities including, hypertension, migraine, pain disorders, hyperlipidaemia, fatigue and sleep disorders, diabetes mellitus, obesity, osteoarthritis, anxiety, chronic pulmonary disease, asthma, depression, cancer, dementia, epilepsy, bipolar disorder, thyrotoxicosis, and alcohol use [Supplementary Table 3; adapted from Dai et al. (21)]. Baseline comorbidities and severity indices were identified based on the presence of one inpatient and/or two confirmed outpatient diagnoses during the 12-month period before index.

#### 2.3.2 Treatment patterns

Treatment use of defined pharmacological therapies and medical interventions was investigated during patient follow-up (Supplementary Table 4). Pharmacological therapies were identified based on the presence of at least one prescription during follow-up; agents were identified using Anatomical Therapeutic Chemical (ATC) in the outpatient setting and German operations and procedure (OPS) codes for the inpatient setting. Therapies identified from visits to services (occupational therapy and physiotherapy) were only available in the AOK dataset and were identified using remedy codes. Treatment pattern analyses were based on an algorithm that included pharmacological therapies only. The list of pharmacological therapies was compiled through a mixture of secondary literature reviews as well as interviews with treating physicians. The list was then expanded to ensure multiple drugs of the same class were included. The first prescription after the initial ET diagnosis marked the start of the first line of therapy. Any subsequent agent prescribed within 30 days of the initial agent was classified as combination therapy if the first observed agent of the combination was also reapplied within 90 days. A subsequent line of therapy was defined if a new agent and/or combination was prescribed. The end of a line of therapy was marked by discontinuation (defined as a 90-day gap after the presumed end of drug supply) of all agents in the prior line or switch to a new agent/combination. The date of discontinuation was defined as the start of the 90-day gap or prescription date of the new agent/combination, whichever came earlier. The date of the switch was the date of the first prescription of the subsequent line of therapy.

### 2.4 Statistical analysis

Point prevalence for each calendar year (2010–2021) was calculated as the ratio of the number of prevalent patients (new and existing cases) with ET to the total number of individuals insured by the sickness fund on January 1 of the respective calendar year. The prevalent cohort considered all individuals who were alive and insured by the sickness fund on January 1 of the respective calendar year and had at least one inpatient and/or two confirmed outpatient diagnoses of ET in two different quarters between January 1, 2010 and December 31 of the year under consideration. Cumulative incidence for each calendar year (2011–2021) was defined as the number of patients newly diagnosed with ET in the respective assessment year divided by the number of insured individuals at risk at the beginning of the respective year, excluding those prevalent in the prior year. Estimates were calculated overall and stratified by age group and gender. Standardized estimates were calculated using the age and gender distribution of the German national population (German Federal Statistical Office). All epidemiological estimates are presented per 100,000 persons.

We used descriptive statistics to summarize baseline characteristics and overall treatment use, including means, standard deviations and medians for continuous variables, as well as frequencies and percentages for categorical variables. Time-to-event outcomes were analyzed using Kaplan–Meier (KM) methodology. Median time-to-event with corresponding 95% confidence intervals (CI) were provided.

Statistical analyses were carried out separately for the AOK and GWQ datasets as data could not be combined at the patient level due to the regulations of the sickness funds. The analyses were conducted using STATA 17 (22).

## 3 Results

### 3.1 Epidemiology

Standardized point prevalence and cumulative incidence increased steadily over the study period in both datasets (Table 1). Point prevalence increased from 32.5 in 2010 to 195.7 per 100,000 persons in 2021 in AOK and 44.7 in 2010 to 249.5 per 100,000 persons in 2021 in GWQ. Cumulative incidence ranged from 10.7 in 2010 to 24.8 per 100,000 persons in 2021 (AOK) and 13.8 in 2010 to 25.7 per 100,000 persons in 2021 (GWQ). Crude prevalence and cumulative incidence estimates showed a similar trend in the overall population (Supplementary Table 5). When stratified by gender and age groups, crude prevalence and cumulative incidence exhibited a tendency to increase with age in both males and females, peaking between ages 70 and 79 years (2021, Supplementary Table 6).

**Table 1:** Point prevalence and cumulative incidence of ET per 100,000 persons between 2010 and 2021 standardized by age and sex to the German national population.

	Standardized prevalence per 100,000 persons (95% CI)		Standardized cumulative incidence per 100,000 persons (95% CI)	
Calendar year	AOK	GWQ	AOK	GWQ
2010	32.5 (32.1–32.89)	44.7 (44.2–45.1)	–	–
2011	42.0 (41.6–42.5)	57.9 (57.4–58.5)	10.7 (10.5–10.9)	13.8 (13.6–14.1)
2012	51.0 (50.5–51.5)	63.0 (62.4–63.5)	10.8 (10.6–11.0)	11.7 (11.5–12.0)
2013	61.7 (61.1–62.2)	78.0 (77.4–78.6)	11.8 (11.5–12.0)	14.4 (14.2–14.7)
2014	72.7 (72.1–73.3)	93.7 (93.0–94.3)	12.3 (12.1–12.5)	13.8 (13.5–14.1)
2015	84.5 (83.9–85.1)	109.2 (108.5–109.9)	14.2 (13.9–14.4)	17.5 (17.2–17.8)
2016	102.6 (101.9–103.3)	131.7 (130.9–132.5)	15.3 (15.1–15.6)	18.0 (17.7–18.3)
2017	110.4 (109.7–111.1)	134.1 (133.3–134.9)	17.8 (17.5–18.1)	17.6 (17.4–17.9)
2018	127.8 (127.0–128.5)	158.1 (157.2–158.9)	17.8 (17.5–18.1)	18.6 (18.3–18.9)
2019	153.1 (152.3–154.0)	186.7 (185.8–187.7)	22.3 (22.0–22.6)	21.3 (21.0–21.6)
2020	168.2 (167.4–169.1)	212.4 (211.4–213.4)	20.0 (19.7–20.3)	19.6 (19.3–19.9)
2021	195.7 (194.8–196.7)	249.5 (248.4–250.6)	24.8 (24.5–25.1)	25.7 (25.3–26.0)

### 3.2 Newly diagnosed ET cohort description

A total of 18,517 (AOK) and 16,796 (GWQ) patients diagnosed with ET between January 1, 2011 and March 31, 2021 were identified. Of these, 4,568 (AOK) and 5,538 (GWQ) patients met the inclusion criteria for newly diagnosed ET (Figure 1).

The newly diagnosed patient cohort was followed for a mean 56.3 months in AOK and 61.8 months in GWQ, respectively (Table 2). The mean age at initial ET diagnosis was older in AOK (61.3 years) compared to GWQ (52.5 years), with approximately similar distribution of males and females (AOK: 50.7% male; GWQ: 55.7% male). The most frequently observed comorbidities were pain disorders (AOK: 69.1%; GWQ: 65.4%), hypertension (AOK: 64.5%; GWQ: 43.6%), and hyperlipidaemia (AOK: 34.8%; GWQ: 29.5%). Notably, pain disorders included a wide array of indications from headaches and migraine to injuries to the nerves (Supplementary Table 3). Clinically diagnosed alcohol use was present in approximately 5.4% (AOK) and 3.7% (GWQ) of patients. Most patients in AOK were employed (27.6%) or retired (55.7%), whereas patients in GWQ were predominantly employed (52.3%) and relatively fewer were retired (13.4%). A higher proportion of patients in AOK experienced all-cause death during the follow-up compared to GWQ (14.6% vs. 7.0%).

**Table 2:** Baseline characteristics of newly diagnosed patients with ET in AOK and GWQ.

Characteristic	AOK	GWQ
Total sample size, n	4,568	5,538
Index year, n (%)		
2011	309 (6.8)	358 (6.5)
2012	319 (7.0)	428 (7.7)
2013	336 (7.4)	453 (8.2)
2014	344 (7.5)	419 (7.6)
2015	391 (8.6)	561 (10.1)
2016	441 (9.7)	581 (10.5)
2017	527 (11.5)	628 (11.3)
2018	545 (11.9)	671 (12.1)
2019	712 (15.6)	754 (13.6)
2020	616 (13.5)	659 (11.9)
2021	28 (0.6)	26 (0.5)
Age at diagnosis, mean (SD) | median	61.33 (18.2) | 65	52.52 (19.6) | 56
Male, n (%)	2,316 (50.7)	3,083 (55.7)
CCI, mean (SD) | median	1.98 (2.4) | 1	2.42 (2.83) | 2
Elixhauser Comorbidity Index, mean (SD) | median	4.57 (7.8) | 2	2.98 (6.60) | 0
CHA_2_DS_2_-VASc Index, mean (SD) | median	2.53 (1.8) | 2	1.41 (1.2) | 1
Comorbidities^a^, n (%)		
Anxiety	686 (15.0)	918 (16.6)
Asthma	483 (10.6)	608 (11.0)
Bipolar disorder	14 (0.3)	21 (0.4)
Cancer (malignant)	560 (12.23)	545 (9.8)
Chronic pulmonary disease	228 (5.0)	375 (6.8)
Dementia	148 (3.2)	71 (1.3)
Depression	1,038 (22.7)	1,339 (24.2)
Diabetes mellitus	1,242 (27.2)	806 (14.6)
Epilepsy	165 (3.6)	131 (2.4)
Fatigue and sleep-related disorders	601 (13.2)	732 (13.2)
Hyperlipidaemia	1,588 (34.8)	1,632 (29.5)
Hypertension	2,945 (64.5)	2,412 (43.6)
Migraine	260 (5.7)	372 (6.7)
Obesity	840 (18.4)	741 (13.4)
Osteoarthritis	1,488 (32.6)	1,280 (23.1)
Pain disorders	3,154 (69.1)	3,622 (65.4)
Thyrotoxicosis	58 (1.3)	47 (0.9)
Alcohol use^b^, n (%)	245 (5.4)	205 (3.7)
Insurance/Employment status^c^, n (%)		
Employed	1,259 (27.6)	2,898 (52.3)
Retirement applicant	5 (0.1)	0 (0.00)
Retired	2,542 (55.7)	742 (13.4)
Unemployed	383 (8.4)	0 (0.0)
Self-payer	157 (3.4)	1,898 (34.3)
Rehabilitant	6 (0.1)	0 (0.0)
Insured family member without own income	216 (4.7)	0 (0.0)
Follow-up characteristics		
Months of follow-up, mean (SD) | median	56.28 (33.8) | 50.0	61.77 (33.6) | 56.7
Death during follow-up, n (%)	666 (14.6)	388 (7.0)

CCI, Charlson comorbidity index; SD, standard deviation.

a Based on the presence of ICD-10-GM codes in the 12-month period before index (ET diagnosis), defined in Supplementary Table 3 (21).

b Based on the presence of ICD-10-GM code: F10.

c Employed: in full or part-time employment; retirement applicant: applied for retirement; retired: in retirement; unemployed: not employed; self-payer: insurance fees paid by the individual (e.g., students, entrepreneur); rehabilitant: fee paid by the “Rentenkasse” (applicable only for the period of rehabilitation stays); insured family member without own income: co-insured family member (e.g., child, housewife, husband/wife during parental leave).

### 3.3 Treatment patterns

#### 3.3.1 Pharmacological therapies and medical interventions during follow-up

Pharmacological therapies and medical interventions used after initial ET diagnosis during the entire follow-up are described in Table 3. Approximately 73.3% (AOK) and 63.6% (GWQ) of patients were treated with at least one of the investigated pharmacological therapies after initial diagnosis. The most frequently observed agents were beta-blockers, particularly propranolol (AOK: 43.8%; GWQ: 49.5%), bisoprolol (AOK: 26.5%; GWQ: 24.2%), and metoprolol (AOK: 27.1%, GWQ: 22.8%). Anticonvulsants and antidepressants were also frequently prescribed, most commonly primidone (AOK: 21.0%; GWQ: 13.0%), pregabalin (AOK: 11.6%; GWQ: 10.2%), and mirtazapine (AOK: 13.3%; GWQ: 15.4%). Medical procedures such as deep brain stimulation, chemodenervation, and MRI-guided focused ultrasound thalamotomy were rarely observed in this study population (<0.5%), whereas 62.7% (AOK) of patients underwent physiotherapy (data not available for GWQ).

**Table 3:** Frequencies of therapies used among newly diagnosed patients any time after initial ET diagnosis in AOK and GWQ^a^.

Therapy	AOK *N* = 4,568	GWQ *N* = 5,538
Pharmacological therapies^b^ *n* (%)		
Any pharmacological therapy	3,348 (73.3)	3,523 (63.6)
Beta-blockers		
Propranolol	1,465 (43.8)	1,744 (49.5)
Metoprolol	908 (27.1)	803 (22.8)
Bisoprolol	887 (26.5)	852 (24.2)
Atenolol	19 (0.6)	31 (0.9)
Sotalol	7 (0.2)	8 (0.2)
Pindolol	1 (0.0)	1 (0.0)
Nadolol	0 (0.0)	0 (0.0)
Anticonvulsants		
Primidone	704 (21.0)	458 (13.0)
Pregabalin	388 (11.6)	360 (10.2)
Gabapentin	317 (9.5)	279 (7.9)
Levetiracetam	87 (2.6)	77 (2.2)
Topiramate	69 (2.1)	100 (2.8)
Zonisamide	11 (0.3)	4 (0.1)
Perampanel	3 (0.1)	4 (0.1)
Antidepressants		
Mirtazapine	444 (13.3)	543 (15.4)
Trazodone	26 (0.8)	52 (1.5)
Calcium channel blockers		
Verapamil	74 (2.2)	56 (1.6)
Nifedipine	65 (1.9)	79 (2.2)
Nimodipine	0 (0.0)	0 (0.0)
Benzodiazepines		
Clonazepam	68 (2.0)	40 (1.1)
Alprazolam	54 (1.6)	51 (1.5)
Antipsychotics		
Olanzapine	37 (1.1)	52 (1.5)
Clozapine	8 (0.2)	9 (0.3)
Carbonic anhydrase inhibitors		
Acetazolamide	19 (0.6)	26 (0.7)
Methazolamide	0 (0.0)	0 (0.00)
Botulinum toxins (A)		
AbobotulinumtoxinA	3 (0.1)	10 (0.3)
IncobotulinumtoxinA	0 (0.0)	0 (0.00)
OnabotulinumtoxinA	0 (0.0)	0 (0.00)
Medical procedures		
Deep brain stimulation	9 (0.2)	6 (0.1)
Chemodenervation	1 (0.02)	4 (0.1)
MRI-guided focused ultrasound thalamotomy	0 (0.0)	1 (0.02)
Other interventions^c^		
Patients receiving physiotherapy	2,862 (62.7)	–
Patients receiving occupational therapy	306 (6.7)	–

a Mean follow-up (SD): 56.3 (33.8) months in AOK and 61.8 (33.6) months in GWQ.

b Agents may be used as monotherapy or as part of a combination therapy.

c Based on a record of at least one visit to the service.

– Estimates available for AOK only.

#### 3.3.2 Pharmacological therapy use in the first line

Using an algorithm to identify monotherapy and combination treatment sequences, the most frequent pharmacological therapies received as a first-line approach are shown in Table 4. Most patients received monotherapy in the first line (AOK: 93.3%; GWQ: 94.3%). The most common first-line therapy approaches were propranolol (AOK: 33.1%; GWQ: 39.5%), followed by metoprolol (AOK: 17.3%; GWQ: 15.5%), and bisoprolol (AOK: 15.4%; GWQ: 15.5%). Among patients who received combination therapy, the most frequently observed combination was mirtazapine + propranolol (AOK: 0.7%; GWQ: 0.8%).

**Table 4:** Frequencies of pharmacological therapies received among newly diagnosed patients in the first line after initial ET diagnosis in AOK and GWQ.

Therapy	AOK *N* = 4,568	GWQ *N* = 5,538
Pharmacological therapies received in the first-line, n (%)		
Any	3,348 (73.3)	3,495 (63.1)
Monotherapy	3,122 (93.3)	3,297 (94.3)
Combination therapy	226 (6.8)	198 (5.7)
Top 10 most frequent therapies received in the first line^a^, n (%)		
Propranolol	1,109 (33.1)	1,381 (39.5)
Metoprolol	580 (17.3)	543 (15.5)
Bisoprolol	517 (15.4)	543 (15.5)
Primidone	372 (11.1)	196 (5.6)
Mirtazapine	143 (4.3)	226 (6.5)
Pregabalin	125 (3.7)	134 (3.8)
Gabapentin	103 (3.1)	72 (2.1)
Topiramate	–	34 (1.0)
Levetiracetam	36 (1.1)	33 (1.0)
Verapamil	35 (1.1)	–
Nifedipine	25 (0.8)	–
Mirtazapine + Propranolol	–	29 (0.8)
Most frequent combination therapies received in the first line, n (%)		
Mirtazapine + Propranolol	24 (0.7)	29 (0.8)
Primidone + Propranolol	17 (0.5)	–
Metoprolol + Primidone	17 (0.5)	–
Pregabalin + Propranolol	17 (0.5)	14 (0.4)
Bisoprolol + Primidone	14 (0.4)	8 (0.2)
Gabapentin + Propranolol	10 (0.3)	9 (0.3)
Bisoprolol + Mirtazapine	8 (0.2)	8 (0.2)
Gabapentin + Metoprolol	8 (0.2)	–
Mirtazapine + Pregabalin	7 (0.2)	–
Bisoprolol + Propranolol	6 (0.2)	8 (0.2)
Metoprolol + Mirtazapine	6 (0.2)	6 (0.2)
Metoprolol + Propranolol	–	8 (0.2)
Mirtazapine + Primidone	–	7 (0.2)
Bisoprolol + Gabapentin	–	6 (0.2)
Metoprolol + Pregabalin	–	6 (0.2)
Olanzapine + Propranolol	–	6 (0.2)
Other combinations (≤5 patients)	92 (2.8)	83 (2.4)

a Includes therapies used as monotherapy or as part of a combination.

– Estimate not applicable.

#### 3.3.3 Time-to-event outcomes

**Figure 2:**
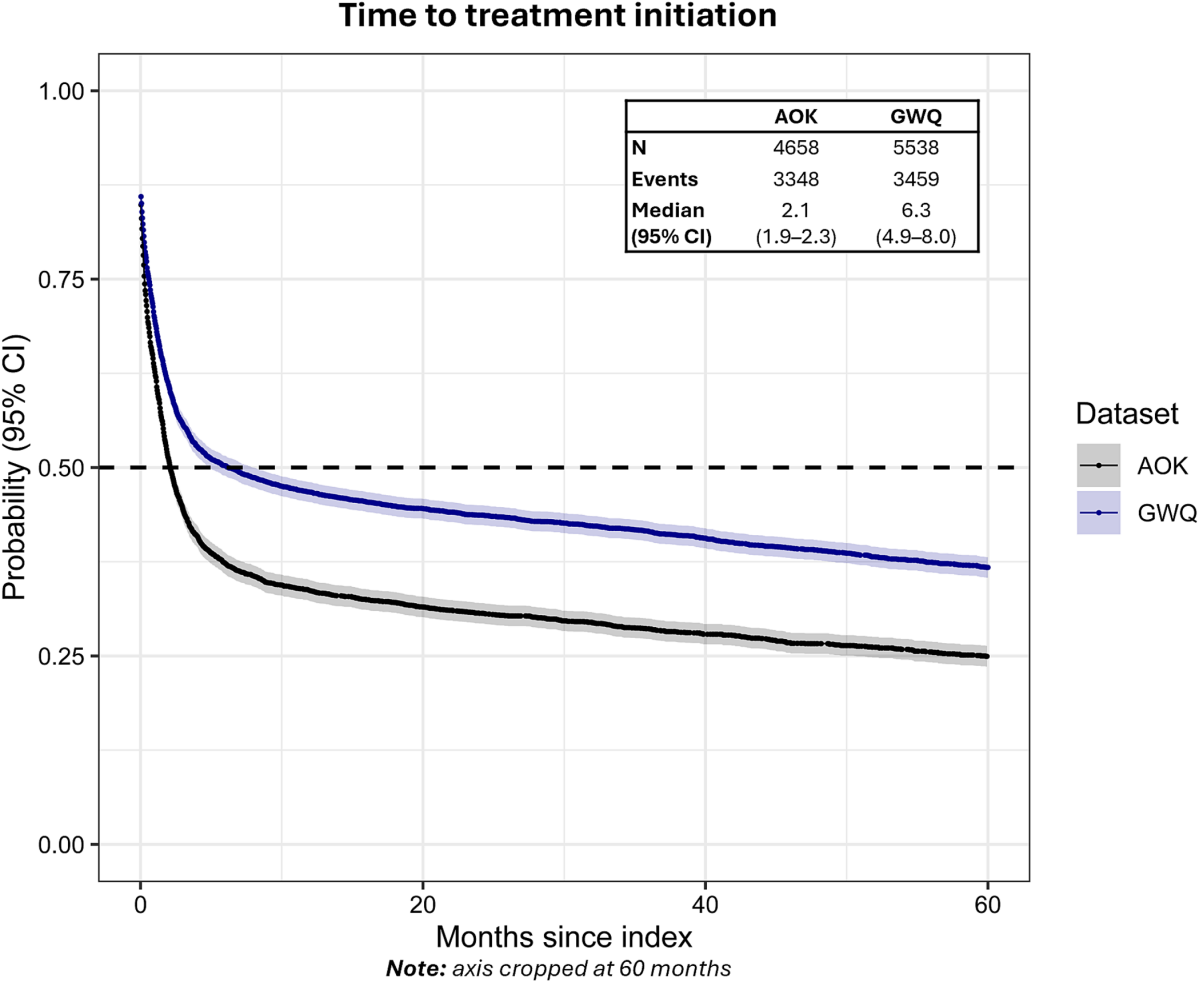
Time to treatment initiation from first ET diagnosis in AOK and GWQ.Dashed line reflects median time-to-event. Gray shading reflects 95% CI.

Among all newly diagnosed patients, 68.4% (*n* = 3,126, AOK) and 56.0% (*n* = 3,102, GWQ) received therapy within 24 months after initial diagnosis. The median time from initial ET diagnosis to the start of therapy was longer in GWQ (6.3 months, 95% CI: 4.9–8.0) compared to AOK (2.1 months, 95% CI: 1.9–2.3) (Figure 2).

**Figure 3:**
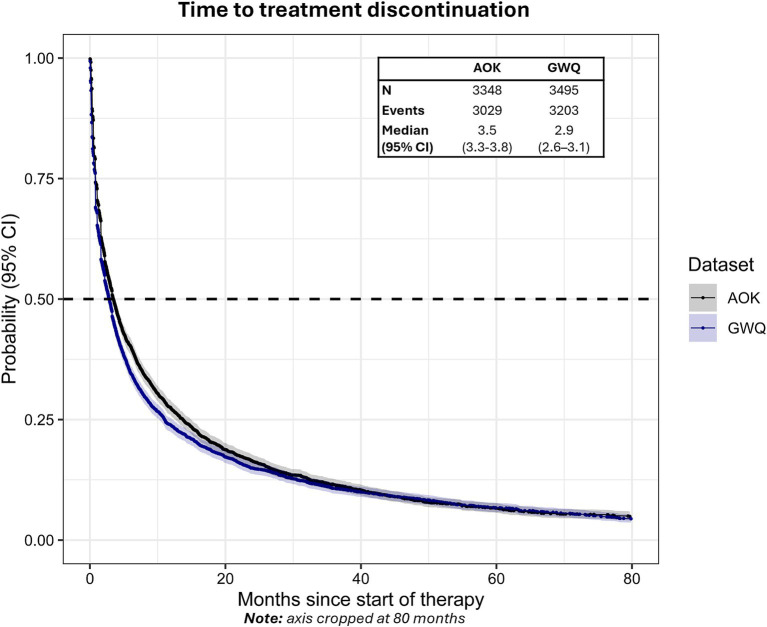
Time to treatment discontinuation from the start of first-line therapy in AOK and GWQ.Dashed line reflects median time-to-event. Gray shading reflects 95% CI.

The median time to discontinuation of first-line therapy was 3.5 months (95% CI: 3.3–3.8) in AOK and 2.9 months (95% CI: 2.6–3.1) in GWQ (Figure 3). Approximately 71.9% (*n* = 2,408, AOK) and 75.4% (*n* = 2,634, GWQ) of patients discontinued first-line therapy within 12 months, whereas 81.9% (*n* = 2,742, AOK) and 83.6% (*n* = 2,921, GWQ) discontinued within 24 months.

**Figure 4:**
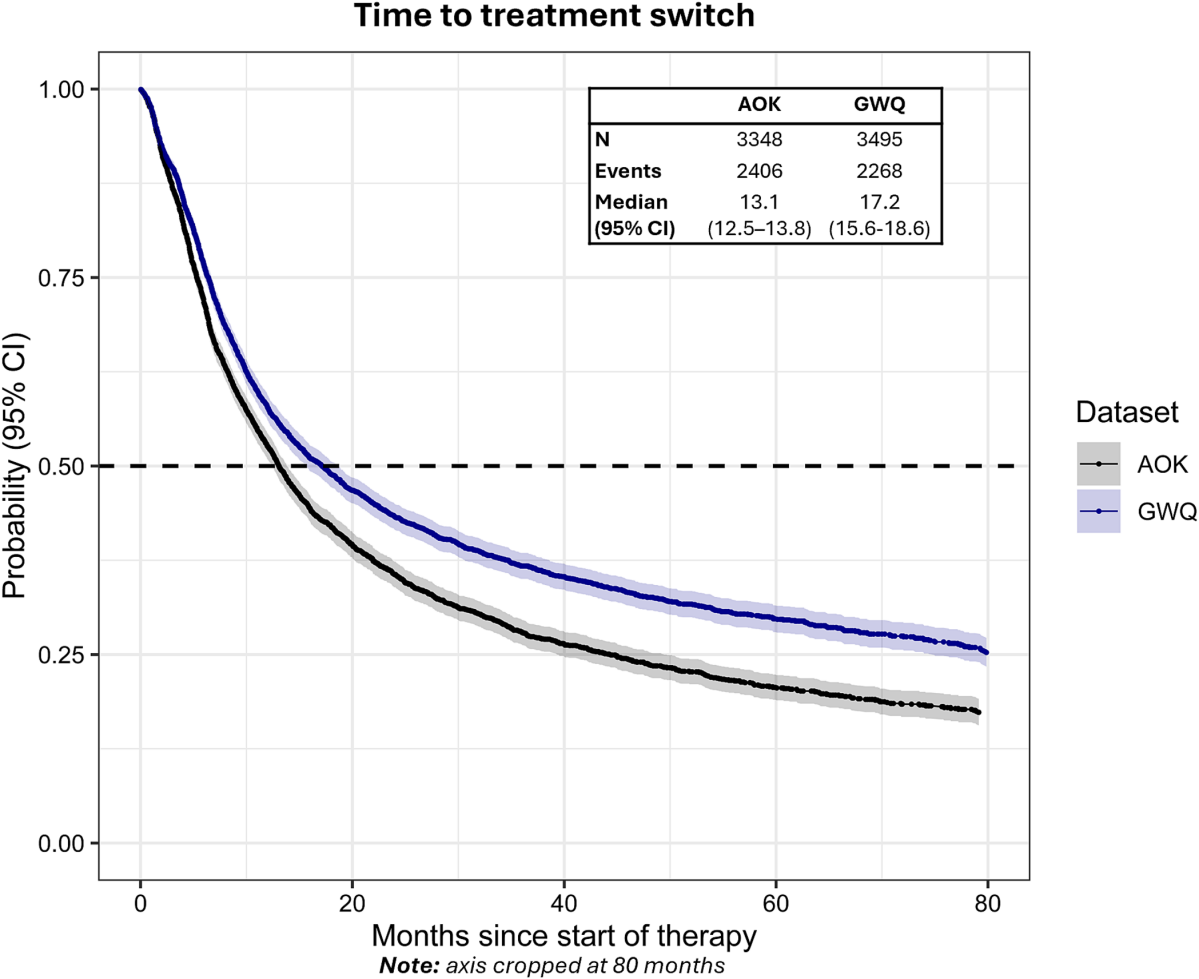
Time to treatment switch from the start of first-line therapy in AOK and GWQ.Dashed line reflects median time-to-event. Gray shading reflects 95% CI.

The median time to treatment switch from the start of first-line therapy was 13.1 months (95% CI: 12.5–13.8) in AOK and 17.2 months (95% CI: 15.6–18.6) in GWQ (Figure 4). Approximately 46.1% (*n* = 1,542) of patients in AOK and 40.9% (*n* = 1,430) of patients in GWQ switched to a second line of therapy within 12 months, with 61.0% (AOK) and 53.7% (GWQ) switching to second line after 24 months.

## 4 Discussion

This study provides evidence on the epidemiology, patient characteristics, and treatment patterns of ET based on the real-world setting in Germany. To our knowledge, this is the first study of the epidemiology of ET in Germany. We observed an increase in the point prevalence and cumulative incidence of ET between 2010 and 2021. While this may reflect a true increase in the manifestation of ET, other possible factors such as a growing understanding and awareness of ET among physicians in the real world may have contributed to this observation. The increased incidence may also reflect a rise in help-seeking behaviors among patients.

The prevalence estimates in this study were lower than other contemporaneous epidemiological estimates for ET reported in alternative settings. A recent systematic review of 29 studies from 13 countries estimated an overall diagnosed prevalence of approximately 320 per 100,000 in 2020 (23). For the same year, we estimated a standardized prevalence of 170 and 212 per 100,000 for AOK and GWQ, respectively. The lower prevalence observed in our study may reflect the stringent definition that we employed, whereby we required at least two confirmed diagnoses in the outpatient setting and excluded patients with several other relevant differential diagnoses. As a result, our study population is likely to exclude patients who have been initially misdiagnosed with ET, resulting in a more homogeneous group of patients and potentially lower prevalence estimates compared to other reports. Notably, while the systematic review found substantial heterogeneity across constituent studies, the nature of the studies included in the meta-analysis differed, with the diagnosis of ET confirmed by a neurologist in most cases (23). The present analysis was based on inpatient or outpatient diagnostic codes observed in health insurance claims made by any physician specialty. In a sensitivity analysis exploring the diagnosing specialty among patients with the index diagnosis in the outpatient setting, the most frequently observed diagnosing specialty was indeed neurology (>48% for the first and >43% for the second confirmatory diagnosis required to meet the outpatient criteria; data not shown). Restricting the diagnosis to those documented by a neurology specialty may have further increased the specificity of our ET definition.

While ET can manifest at any age, prior research has shown that prevalence tends to peak in the sixth decade of life, with a slightly higher prevalence in males across all age groups (23). Our findings showed a sharp increase in prevalence and incidence in the sixth decade of life, peaking for the 70–79 age group. Interestingly, for GWQ but not AOK, we also observed a second, lower peak at the 40–49 age group.

The diagnosis of ET remains challenging (24), which contributes to the difficulty in accurately and consistently estimating its prevalence and incidence. One significant obstacle is the clinical heterogeneity of the disease, as individuals may exhibit a wide range of symptoms and severity levels (25, 26). This diversity makes it challenging to identify consistent biomarkers or establish clear diagnostic criteria. Additionally, the overlap in symptoms between ET and other neurological disorders, such as Parkinson’s disease, further complicates diagnosis and classification. Consensus criteria for classifying ET were published by the International Parkinson and Movement Disorder Society in 1998 and updated in 2018 (27, 28). The most recent consensus guidelines consider ET to be a family of disorders and differentiate ET from ET *plus* (patients meeting the criteria for ET with additional neurological signs of uncertain significance) (27). Given that ET *plus* is not yet a separate diagnosis in the ICD-10 classification, and this term is still debated in clinical practice, our study does not provide information about the epidemiology of specific ET subtypes. Substantial improvements in the understanding of ET and changes in consensus criteria for its classification may partly explain the changes in incidence and prevalence over time and heterogeneity across studies.

Some differences in patient profiles and outcomes were observed between the AOK and GWQ datasets. Compared with GWQ, patients in AOK were much more likely to be retired and comorbid as indicated by higher mean Elixhauser comorbidity scores (4.6 vs. 3.0) and CHA_2_DS_2_-VASc index (2.5 vs. 1.4), although mean CCI scores were rather similar (2.0 vs. 2.4). In addition, a higher proportion of patients in AOK received pharmacological therapy (73% vs. 64%). Most notably, the median time from initial ET diagnosis to treatment start was considerably longer in GWQ than in AOK (2.1 vs. 6.3 months). These differences are likely explained by the fact that, on average, patients in the AOK cohort were older than in GWQ (median age 57 vs. 65 years). Prior evidence has shown that older age groups tend to be slightly overrepresented in AOK compared with the national population, aligning with the differences observed between the two cohorts (29).

Our study corroborates emerging evidence for high comorbidity burden in ET and the presence of non-motor features. Consistent with another study using a large US-based administrative claims database (21), we observed that pain disorders (>65%), hypertension (44–65%), and hyperlipidaemia (30–35%) were among the most frequently comorbid conditions. Moreover, our study aligns with extensive evidence suggesting that patients affected by ET may also suffer increased psychiatric disorders (30), with approximately 15–25% of patients receiving a diagnosis of depression or anxiety. Notably, many of the comorbid conditions observed among the cohorts are known to be age-related. ET onset typically occurs at a more advanced age; therefore, it is also possible that the presence of these conditions may be more closely related to age than ET specifically.

Guidelines from the International Movement Disorder Society (MDS) for the treatment of ET, as well as guidelines for Germany, recommend a combination therapy of propranolol with primidone or topiramate, particularly in the first line (8, 31). Metoprolol is additionally indicated, given its frequent use for cardiological indications (31). The most frequently prescribed medications in our study broadly align with treatment guidelines. However, very few patients were prescribed topiramate despite its recommendation in guidelines. Furthermore, a variety of other medications were observed among the cohort, which are not recommended pharmacological treatments for ET. This may be reflective of the prevalence of concomitant comorbidities observed in the cohort. For example, mirtazapine was frequently prescribed and may have benefits for reducing ET symptoms in addition to addressing anxiety and depression, which were frequent comorbid conditions in the presented populations.

Among the newly diagnosed cohort, monotherapy was used more frequently than combination therapy. However, this may be a limitation of our study, given that the data cannot account for nuances of treatment use in the real world. For example, beta-blockers are frequently prescribed for patients to use “as needed”, and the prescribed dosage does not always accurately reflect how a patient uses the medication on a daily basis. Furthermore, our algorithm for combination therapies included agents prescribed within a 30-day window of another. It is possible that this duration of overlap was too short to capture combination therapies with high sensitivity. In a clinical setting, add-on therapies may be prescribed approximately three months after starting the initial agent. Therefore, our study may have misclassified some therapies as monotherapies, although they were intended to be used as add-ons.

Generally, high-quality evidence for the effectiveness of therapeutic options beyond beta-blockers, primidone, and topiramate is limited (31, 32). Moreover, there is a notable absence of clear evidence for effective therapies beyond the first line. The high discontinuation and treatment switch rates observed in our study may be the result of a tendency for patients treated with first-line therapies (propranolol and primidone) to develop tolerance to drug effects and the potential to experience adverse effects when using these drugs in the long term (33). This is also in line with findings from a UK/French primary care study (5) and another US-based study, which similarly showed high rates of therapy discontinuation (34). We additionally observed that approximately 30% of patients with ET in our study did not use any pharmacotherapy following initial diagnosis. Our findings align with evidence from a prior US-based claims data study, which similarly showed that only approximately 60% of patients with ET receive pharmacological treatment after diagnosis (35). In some cases, patients may have had mild tremors decidedly not severe enough to warrant pharmacological intervention or impactful enough to warrant tolerating treatment side effects, particularly given the relatively poor response rate. However, our findings may also suggest considerable undertreatment of ET symptoms.

Preliminary evidence suggests that very few patients with ET are satisfied with their care (36). A great deal more evidence for patient preferences in ET is needed to better understand the low levels of pharmacological therapy use and non-persistence among patients to improve patient care and, ultimately, patient outcomes. Given a lack of evidence-based options for ET treatment, clinicians must often weigh a number of factors together with the patient, in a “trial and error” approach. There are several pharmacotherapies currently in development that show promise for improving outcomes in ET, such as T-type calcium channel modulators, CB-1 agonists, and GABA A receptor-positive allosteric modulators (37).

Moreover, advances in medical interventions, such as deep brain stimulation and MRI-guided focused ultrasound, have shown effectiveness in patients with medication refractory ET (37–40). Notably, a low proportion of patients underwent medical procedures such as deep brain stimulation, chemodenervation, and MRI-guided focused ultrasound thalamotomy in this patient population. The use of these procedures is likely expected to be concentrated in tertiary neuroscience centers or larger academic hospitals, and therefore, most clinicians treating ET (e.g., in primary care) may not be using these treatments and may have a very high threshold for referral due to perceptions of invasiveness. While MRI-guided focused ultrasound thalamotomy is non-invasive compared to deep brain stimulation, its availability has increased in recent years, and uptake may not yet adequately be reflected in our study period.

The foremost strength of this study was the use of German SHI data, which provided a large representative sample of patients with ET covering all relevant inpatient and outpatient care. Given that over 85% of the German population is insured through SHI, the data used in this study reflect representative healthcare regulations and practices across public health insurance companies in Germany. Our study employed a stringent definition of ET to reduce the risk of misclassification. Finally, the availability of long follow-up data allowed for a longitudinal assessment of treatment patterns, with over 50% of patients with ET followed for 50 months or longer.

While rigorous, our study only captured patients with insurance claims for ET, and therefore, some cases of mild tremor or patients who did not seek a diagnosis may have been missed. Due to the nature of the secondary anonymized data used in this study, the diagnosis for ET was defined using ICD-10-GM codes documented by any specialty. In a validation study conducted in the US, the ICD-10-CM (clinical modification) for ET exhibited only a 74.4% positive prediction value in identifying true ET (41). As such, ICD-10 diagnoses recorded in insurance claims may be subject to misclassification without additional information from clinical notes and validation from a neurologist. Despite these limitations, the coding of these databases in Germany is subject to quality checks by health insurance funds due to direct relevance for reimbursement, particularly in the inpatient setting, and is generally considered to be of high quality for epidemiological research (42–44). Moreover, to define the ET cohorts in this study, patients with prescriptions for tremor-inducing agents including valproate, lithium, cyclosporin, and tacrolimus in the 12-month period before the ET diagnosis or select tremor-related conditions after ET diagnosis were excluded. Other conditions or agents such as asthma medications (beta-adrenergic agonists) may also be classified as tremor-inducing, although they were not considered in this study to make a trade-off between stringency to ensure the accuracy of ET classification and avoiding bias by excluding patients with possible concomitant conditions alongside their ET. Furthermore, information on the prescribed frequency of dosage of the pharmacotherapies and treatment adherence is not directly available in the data. As such, we relied on an algorithm to identify treatment discontinuation, and for some therapies, this may have resulted in some misclassification of treatment lines.

## 5 Conclusion

This study provided real-world estimates for the epidemiology, patient characteristics, and treatment patterns of ET using two large representative claims databases in Germany. Although the diagnosis of ET remains a challenge, the understanding and classification of the disease have changed considerably over the past two decades. We used strict criteria based on claims data to define and study a sample of patients with ET. Among these patients, comorbidities were common, particularly pain and cardiovascular conditions. Evidence-based treatments for ET include propranolol and primidone, as well as topiramate and metoprolol, which were frequently used as first-line approaches in our samples. Despite advances, non-pharmacological medical interventions such as MRI-guided focused ultrasound were rarely observed. Overall, there remains a high unmet treatment need in ET, and further evidence, particularly for evidence-based treatments beyond the first line is needed.

## Data Availability

The datasets presented in this article are not readily available because of ethical and privacy restrictions. Requests to access the datasets should be directed to the corresponding author/s.
