# Supplementary material for: Epidemiology and treatment patterns of essential tremor: a retrospective cohort analysis in Germany
Source: Front Neurol. 2025 Jul 2;16:1580919. doi: 10.3389/fneur.2025.1580919 (PMC12265497; doi:10.3389/fneur.2025.1580919)
Supplement: Supplementary file 1 [file Supplementary_file_1.docx]

**Epidemiology and treatment patterns of essential tremor: A retrospective cohort analysis in Germany**

Supplemental Table 1. Charlson Comorbidity Index scoring and ICD-10-GM codes

| **No.** | **Comorbidity** | **Score** | **ICD-10-GM codes** |
| --- | --- | --- | --- |
| 1 | Coronary artery disease | 1 | I20, I21, I22, I23, I24, I25 |
| 2 | Congestive heart failure | 1 | I11, I50 |
| 3 | Peripheral vascular disease | 1 | I73, I74, I77 |
| 4 | Cerebrovascular disease | 1 | G45, G46, I6 |
| 5 | Dementia | 1 | F00, F01, F02, F03, G30 |
| 6 | Chronic pulmonary disease | 1 | J4x, J6 (except J67, J68, J69) |
| 7 | Connective tissue disorder | 1 | M05, M06, M07, M08, M3 |
| 8 | Peptic ulcer disease | 1 | K25, K26, K27, K28 |
| 9 | Mild liver disease | 1 | B18, K70, K73, K75 |
| 10 | Diabetes mellitus without complications | 1 | E10.9, E11.9, E12.9, E13.9, E14.9 |
| 11 | Hemiplegia | 2 | G81, G82 |
| 12 | Moderate or severe renal disease | 2 | N17, N18, N19 |
| 13 | Diabetes mellitus with end-organ damage | 2 | E10, E11, E12, E13, E14 (except B18, K70, K73, K75) |
| 14 | Tumor without metastases, leukemia, lymphoma, multiple myeloma | 2 | C (Except C77, C78, C79, C80) |
| 15 | Moderate or severe liver disease | 3 | K72, K74, I85 |
| 16 | Metastatic solid tumor | 6 | C77, C78, C79, C80 |
| 17 | AIDS | 6 | B20, B21, B22, B23, B24 |

The CCI scoring was based on (16), adapted for German claims.

Supplemental Table 2. CHA2DS2-VASc scoring and ICD-10-GM codes

| **Risk factors** | | **Points** | **ICD-10-GM codes** |
| --- | --- | --- | --- |
| C | Congestive heart failure | 1 | I50 |
| H | Hypertension | 1 | I10–I15 |
| A | Age at the time of assessment ≥75 years | 2 | - |
| D | Diabetes mellitus | 1 | E10–E14 |
| S | Previous stroke, TIA, or thromboembolism | 2 | G45.9, I63, I74 |
| V | Vascular disease (previous myocardial infarction, coronary artery disease, peripheral arterial occlusive disease, atherosclerosis of the aorta) | 1 | I21, I22, I25.0, I25.1, I25.2, I70.0, I70.2, I73.9 |
| A | Age at the time of assessment between 65 - 74 years | 1 | - |
| Sc | Sex category (female) | 1 | - |
| Maximum score | | 9 |  |

ICD-10-GM codes were defined in accordance with (19, 20)

Supplemental Table 3. ICD-10-GM codes used to identify comorbidities

| **Comorbidity** | **ICD-10-GM codes** |
| --- | --- |
| Hypertension | I10–I13, I15 |
| Migraine | G43 |
| Pain disorders^a,b^ | G04, G05, G36, G37, G43, G44, G50, G51, G54, G56, G57, G58.7, G60, G90, G95, G99, M05, M06, M12, M43, M45, M46, M47, M48, M50, M51, M53, M54, M60, M79, M96.1, R07, R10, R16, R19, R51, R52, F45.4, B02.2, S04.1, S14.1, S24.1, S34.1, S34.3, S44, S54, S64, or S74 |
| Hyperlipidemia^b^ | E78.0–E78.5 |
| Fatigue and sleep-related disorders^b^ | R53.1, R53.8, F48.8, F51.0, F51.1, F51.8, G47, G93.3 |
| Diabetes mellitus^b^ | E10–E14 |
| Obesity^b^ | E66, Z68.3, Z68.4 |
| Osteoarthritis^b^ | M15–M19 |
| Anxiety^b^ | F40, F41, F42, F43.0, F43.1, F43.9, F48.8, F48.9, R45.2, R45.3, R45.4, R45.5, R45.6, R45.7, R45.8, F06.4 |
| Chronic pulmonary disease^b^ | J40, J47, J60, J67, I27.8, I27.9, J68.4, J70.1, J70.3 |
| Asthma | J45 |
| Depression^b^ | F32, F33 |
| Cancer (malignant)^b^ | C00–C97 |
| Dementia^b^ | F00. F03, F05.1, G30, G31.1 |
| Epilepsy | G40 |
| Bipolar disorder | F31 |
| Thyrotoxicosis | E05 |
| Alcohol use | F10 |

[a] Encephalitis, myelitis and encephalomyelitis (G04-G05), Other acute disseminated demyelination (G36-G37), Migraine (G43), Other headache syndromes (G44), Diseases of the trigeminal or facial nerve (G50-G51), Diseases of nerve roots and nerve plexuses (G54), Mononeuropathies of the upper extremities (G56), Mononeuropathies of the lower extremities (G57), Mononeuritis multiplex (G58.7), Hereditary and idiopathic neuropathy (G60), Diseases of the autonomic nervous system (G90), Other diseases of the spinal cord (G95), Other diseases of the nervous system in diseases classified elsewhere (G99),Seropositive chronic polyarthritis and Other chronic polyarthritis (M05-M06), Other specified arthropathies (M12), Other deformities of the spine and back (M43), Ankylosing spondylitis, Other inflammatory spondylopathies, Spondylosis, Other spondylopathies, (M45-M48), Cervical disc damage and Other disc damage (M50-M51), Other diseases of the spine and back, not elsewhere classified (M53), Back pain (M54), Myositis (M60), Other soft tissue disorders, not elsewhere classified (M79), Postlaminectomy syndrome, not elsewhere classified (M96.1), Sore throat and chest pain (R07), Abdominal and pelvic pain (R10), Hepatomegaly and splenomegaly, not elsewhere classified (R16),Other symptoms affecting the digestive system and abdomen (R19), Headache (R51), Pain, not elsewhere classified (R52), Persistent pain disorder (F45.4), Zoster with involvement of other parts of the nervous system (B02.2), Injury to cranial nerves (S04.1), Other and unspecified injuries of the cervical spinal cord (S14.1), Other and unspecified injuries of the thoracic spinal cord (S24.1), Other lumbar spinal cord injury (S34.1), Injury to the cauda equina (S34.3), Injuries to nerves, blood vessels, muscle and tendons including crushing or traumatic amputation of shoulder and upper arm (S44-S49), Superficial injury, open wound, fracture, dislocation or injury to nerve of the forearm (S50-S54), Injury to nerves at the level of the wrist and hand (S64), Injury to nerves at the level of the hip and thigh (S74)

[b] Adapted to ICD-10-GM from (21)

Supplemental Table 4. Codes to identify ET-related pharmacological therapies, non-pharmacological interventions and service-based therapies

| **Therapy** | **Codes** |
| --- | --- |
| **Pharmacological Therapies** |  |
| Beta-blockers |  |
| Atenolol | ATC: C07AB03 |
| Metoprolol | ATC: C07AB02 |
| Nadolol | ATC: C07AA12 |
| Pindolol | ATC: C07AA03 |
| Propranolol | ATC: C07AA05 |
| Bisoprolol | ATC: C07AB07 |
| Sotalol | ATC: C07AA07 |
| Antidepressants |  |
| Mirtazapine | ATC: N06AX11 |
| Trazodone | ATC: N06AX05 |
| Anticonvulsants |  |
| Gabapentin | ATC: N03AX12 |
| Levetiracetam | ATC: N03AX14 |
| Perampanel | ATC: N03AX22 |
| Pregabalin | ATC: N03AX16 |
| Primidone | ATC: N03AA03 |
| Topiramate | ATC: N03AX11 |
| Zonisamide | ATC: N03AX15 |
| Benzodiazepines |  |
| Alprazolam | ATC: N05BA12 |
| Clonazepam | ATC: N03AE01 |
| Antipsychotics |  |
| Clozapine | ATC: N05AH02 |
| Olanzapine | ATC: N05AH03 |
| Carbonic anhydrase inhibitors |  |
| Acetazolamide | ATC: S01EC01 |
| Methazolamide | ATC: S01EC05 |
| Botulinum toxins (A) |  |
| AbobotulinumtoxinA | ATC: M03AX21  OPS: 6-003.8 |
| IncobotulinumtoxinA |  |
| OnabotulinumtoxinA |  |
| Potassium Channel Blockers |  |
| Perampanel | ATC: N03AX22 |
| Calcium Channel Blockers |  |
| Nifedipine | ATC: C08CA05 |
| Nimodipine | ATC: C08CA06 |
| Verapamil | ATC: C08DA01 |
| **Non-pharmacological Interventions** |  |
| Deep brain stimulation | OPS: 8-631 |
| Chemodenervation | Equivalent to ATC/OPS codes for botulinum toxins |
| MRI-guided focused ultrasound thalamotomy | OPS: 5-013.72, 5-014.e |
| **Service-based therapies** |  |
| Occupational Therapy | Heilmittel codes starting with 5 |
| Physiotherapy | Heilmittel codes starting with 2 |

Abbreviations: ATC: Anatomical Therapeutic Chemical; ET: essential tremor; OPS: German operations and procedure code

Supplemental Table 5. Crude prevalence and cumulative incidence of ET per 100,000 persons between 2010 and 2021

|  | **Crude prevalence per 100,000 (95% CI)** | | **Crude cumulative Incidence per 100,000 (95% CI)** | |
| --- | --- | --- | --- | --- |
| **Calendar Year** | **AOK** | **GWQ** | **AOK** | **GWQ** |
| 2010 | 38.56 (36.17-41.08) | 35.68 (33.62-37.83) | - | - |
| 2011 | 48.37 (45.70-51.16) | 46.08 (43.82-48.42) | 12.45 (11.10-13.91) | 11.71 (10.54-12.97) |
| 2012 | 58.61 (55.67-61.68) | 56.07 (53.67-58.56) | 12.62 (11.28-14.09) | 10.85 (9.78-12.00) |
| 2013 | 70.09 (66.86-73.43) | 68.49 (65.83-71.22) | 13.34 (11.95-14.84) | 13.08 (11.93-14.31) |
| 2014 | 81.57 (78.09-85.16) | 82.20 (79.29-85.20) | 13.60 (12.20-15.12) | 12.49 (11.37-13.70) |
| 2015 | 95.79 (92.04-99.65) | 96.84 (93.71-100.06) | 15.80 (14.29-17.42) | 15.94 (14.67-17.29) |
| 2016 | 113.98 (109.93-118.14) | 114.94 (111.52-118.43) | 17.20 (15.63-18.88) | 16.38 (15.10-17.74) |
| 2017 | 120.24 (116.33-124.26) | 127.91 (124.38-131.51) | 19.06 (17.42-20.81) | 17.19 (15.90-18.56) |
| 2018 | 134.78 (130.69-138.95) | 144.90 (141.18-148.70) | 18.57 (17.05-20.19) | 17.83 (16.53-19.21) |
| 2019 | 160.28 (155.82-164.83) | 167.99 (163.97-172.08) | 23.41 (21.72-25.19) | 20.05 (18.67-21.51) |
| 2020 | 172.55 (168.04-177.15) | 186.71 (182.49-191.00) | 20.45 (18.90-22.10) | 18.29 (16.98-19.68) |
| 2021 | 188.80 (184.10-193.59) | 204.24 (199.82-208.73) | 19.03 (17.55-20.60) | 16.03 (14.81-17.33) |

Abbreviations: CI: confidence interval

Supplemental Table 6. Crude prevalence and cumulative incidence of ET per 100,000 per 100,000 persons for 2021, stratified by age group and gender

|  | **Crude prevalence per 100,000 (95% CI)** | | **Crude cumulative Incidence per 100,000 (95% CI)** | |
| --- | --- | --- | --- | --- |
| **Gender / Age group** | **AOK** | **GWQ** | **AOK** | **GWQ** |
| Female |  |  |  |  |
| 0-17 | 8.31 (5.32-12.36) | 150.48 (137.53-164.31) | 3.35 (1.53-6.35) | 16.70 (13.24-20.78) |
| 18-29 | 88.25 (74.09-104.31) | 152.32 (138.47-167.17) | 16.61 (10.75-24.51) | 15.17 (11.85-19.13) |
| 30-39 | 91.63 (79.75-104.80) | 360.77 (336.63-386.17) | 10.15 (6.44-15.24) | 35.20 (30.00-41.04) |
| 40-49 | 106.35 (91.92-122.41) | 420.91 (371.93-474.53) | 12.84 (8.14-19.27) | 32.94 (26.65-40.27) |
| 50-59 | 190.34 (173.17-208.74) | 184.49 (170.75-199.03) | 24.40 (18.48-31.62) | 20.12 (16.44-24.38) |
| 60-69 | 296.72 (275.11-319.57) | 167.34 (152.47-183.26) | 26.13 (19.99-33.57) | 16.32 (12.83-20.45) |
| 70-79 | 426.13 (395.35-458.65) | 496.21 (467.41-526.32) | 59.78 (48.64-72.70) | 58.65 (51.67-66.30) |
| ≥80 | 382.90 (356.06-411.21) | 623.48 (555.76-697.15) | 33.44 (26.21-42.04) | 40.92 (31.90-51.69) |
| Male |  |  |  |  |
| 0-17 | 14.17 (10.26-19.09) | 130.68 (118.63-143.63) | 5.67 (3.24-9.20) | 19.16 (15.41-23.55) |
| 18-29 | 124.94 (108.15-143.60) | 128.20 (115.49-141.92) | 19.86 (13.50-28.19) | 11.94 (8.99-15.54) |
| 30-39 | 104.80 (91.86-119.05) | 307.79 (285.58-331.27) | 9.46 (5.85-14.46) | 39.59 (34.05-45.78) |
| 40-49 | 114.99 (100.14-131.42) | 352.67 (310.20-399.31) | 17.88 (12.31-25.11) | 21.92 (16.92-27.94) |
| 50-59 | 204.51 (186.28-224.04) | 157.73 (145.06-171.22) | 31.25 (24.36-39.48) | 17.78 (14.30-21.86) |
| 60-69 | 382.61 (356.57-410.05) | 146.29 (132.36-161.28) | 55.09 (45.53-66.07) | 11.50 (8.59-15.08) |
| 70-79 | 616.79 (574.67-661.17) | 415.64 (389.49-443.08) | 78.66 (64.28-95.30) | 37.92 (32.35-44.16) |
| ≥80 | 520.13 (477.00-566.09) | 534.83 (475.86-599.05) | 43.15 (32.03-56.89) | 33.49 (25.50-43.20) |

Abbreviations: CI: confidence interval
